# Omega-3-induced xanthoma in a child: A case report

**DOI:** 10.1016/j.jdcr.2025.04.011

**Published:** 2025-04-25

**Authors:** Abdulellah Aleissa, Hadeel Alsulaimani, Waseem Alhawsawi, Sultana Kadasa, Khalid AlHawsawi

**Affiliations:** a King Abdulaziz University, Jeddah, Saudi Arabia; b King Fahad Hospital, Jeddah, Saudi Arabia; c King Fahad Armed Forces Hospital, Jeddah, Saudi Arabia; d University of Jeddah, Jeddah, Saudi Arabia; e Dermatology Department, King Abdulaziz Hospital, Makkah, Saudi Arabia

**Keywords:** drug-induced xanthoma, hypercholesterolemia, internal medicine, medical dermatology, pediatrics, xanthoma

## Introduction

Xanthomas are distinct dermatological signs of lipid metabolism abnormalities, marked by localized lipid deposits in the skin, tendons, and connective tissues, often associated with hyperlipidemia.^1^ While commonly linked to inherited lipid disorders like familial hypercholesterolemia (FH), they can also result from dietary or drug-induced lipid disturbances.^2^ This case highlights a rare instance of pediatric xanthoma caused by excessive omega-3 fatty acid supplementation, a scarcely documented association.

Omega-3 fatty acids, including eicosapentaenoic acid (EPA) and docosahexaenoic acid (DHA), are essential nutrients primarily obtained through diet or supplementation. While generally beneficial for cardiovascular health due to their triglyceride-lowering effects,^3^ certain formulations can paradoxically raise low-density lipoprotein cholesterol (LDL-C).^4^ The impact on lipid metabolism is dose-dependent, with excessive intake potentially disrupting lipid balance.^5^ This effect is more pronounced in pediatric patients due to their distinct metabolic pathways and dosage requirements.^6^

We describe the case of a 7-year-old boy with asymptomatic, slowly progressing bilateral xanthomas of the elbows, knees, popliteal fossae, and palmar creases. By detailing the clinical presentation, diagnostic process, and treatment outcomes, this report aims to contribute to the growing body of evidence on pediatric xanthomas and provide insights into the safe use of omega-3 fatty acid supplements in children.

## Case report

A 7-year-old boy presented with a 2-year history of slowly progressive asymptomatic bilateral skin lesions. His parents reported that for the past 6 months, he had been consuming 3 or more omega-3 fatty acid capsules daily, amounting to 3000-4000 mg per day. The capsules were described as visually appealing, resembling candy, and were used as supplements under the belief that they were beneficial. The patient was not taking any additional medications.

A review of the systems revealed a history of severe chronic headaches, diarrhea, and joint pain. These symptoms were not further evaluated during the initial visit and were not clearly associated with omega-3 fatty acid overconsumption. The patient had a history of lactose intolerance but no family history of this condition.

**Fig 1:**
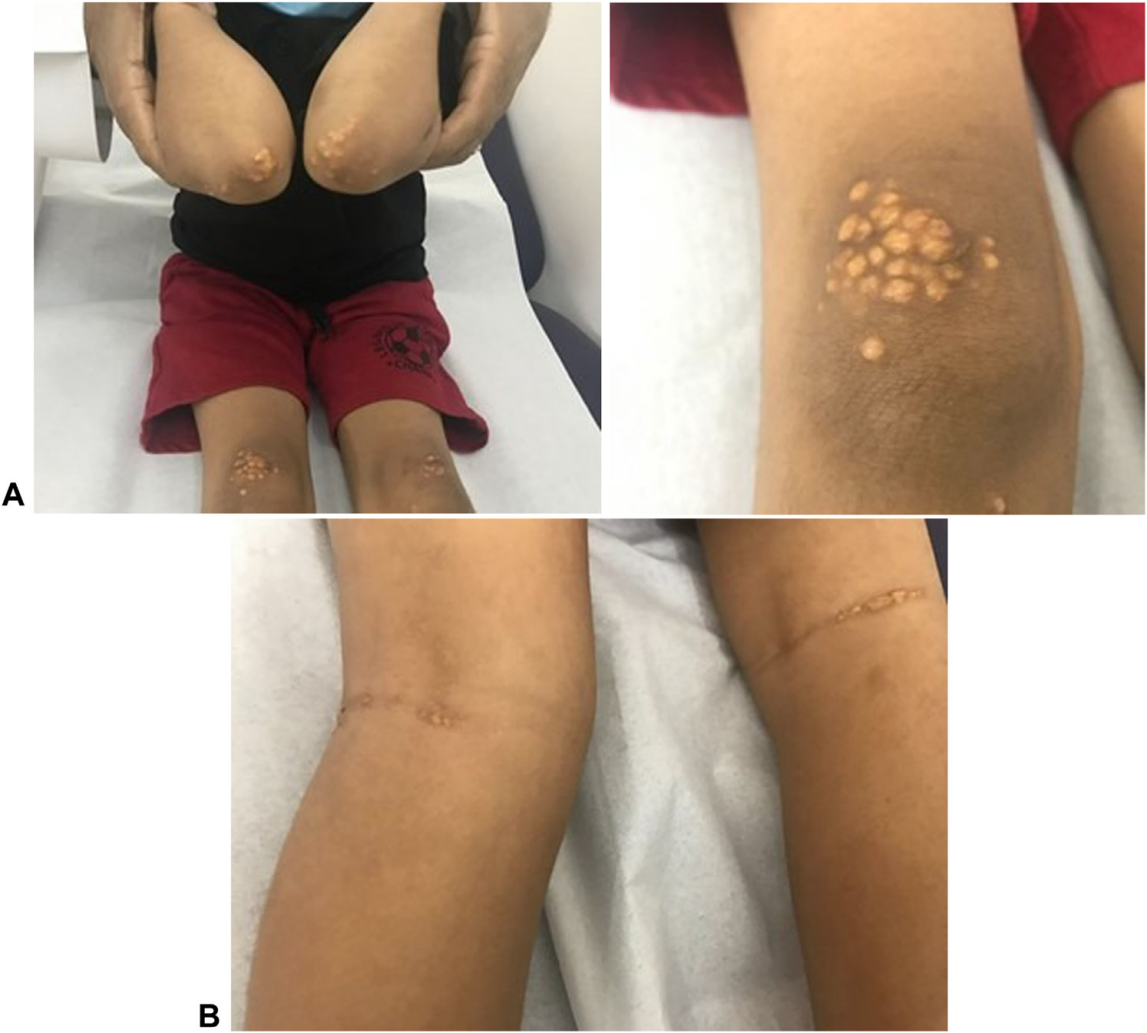
**A,***Yellowish* nodules on the elbows and knees. **B,** Linear *yellow* plaques on the popliteal fossae.

**Fig 2:**
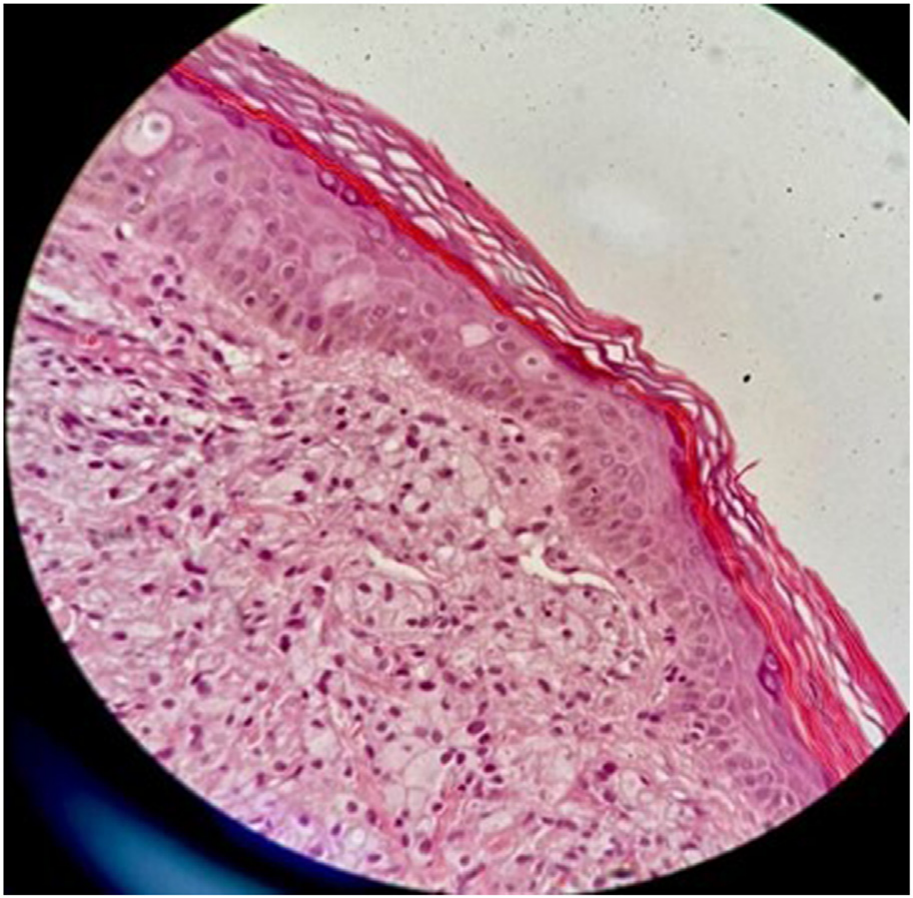
Skin biopsy image showing dense foamy histiocytes throughout the dermis.

On physical examination, multiple nonscaly yellowish nodules were observed bilaterally on the elbows and knees, multiple yellowish linear plaques were found bilaterally on the popliteal fossae (Fig 1, *A* and *B*), and bilateral linear yellow patches were observed along the palmar creases. The hair, nails, and mucous membranes were normal. Skin biopsy revealed dense foamy histiocytes throughout the dermis (Fig 2). Complete blood count, liver function, thyroid function, fasting blood sugar, glycated hemoglobin, serum creatinine, and electrolyte levels were normal. The total cholesterol, LDL cholesterol, and cholesterol/high-density lipoprotein ratio were elevated, whereas very LDL and high-density lipoprotein levels were normal. The results are summarized in Table I.

**Table I:** Fasting lipid profile

Test	Unit	Result	Range
Total cholesterol	mmol/L	22.1 ↑↑	0-5.17
Triglycerides	mmol/L	1.04	0-1.7
HDL-cholesterol	mmol/L	1.53	1.04-1.55
LDL-cholesterol	mmol/L	18.5 ↑↑	0-2.59
Cholesterol/HDL ratio	%	14.44 ↑↑	4-7

*HDL*, High-density lipoprotein; *LDL*, low-density lipoprotein.

The patient was diagnosed with tuberoeruptive (elbows and knees) and plane xanthomas (popliteal fossae and palms) caused by severe hypercholesterolemia secondary to excessive omega-3 fatty acid supplementation. Primary differential diagnoses included FH, type 3 hyperlipoproteinemia, and drug-induced hypercholesterolemia.

Although genetic testing for FH or type 3 hyperlipoproteinemia was not performed, the normalization of lipid levels after discontinuing omega-3 supplementation strongly supported the diagnosis of drug-induced hypercholesterolemia.

The patient was referred to the pediatric endocrinology department for further management. Omega-3 fatty acid supplementation was discontinued, and plasmapheresis was recommended to rapidly reduce cholesterol levels; however, the parents declined the plasmapheresis.

During follow-up, the total cholesterol and LDL levels returned to normal, and the skin lesions resolved, leaving postinflammatory hyperpigmentation within 1 year.

## Discussion

Xanthomas predominantly consist of foam cells, which are macrophages engorged with oxidized LDL particles extravasated from blood vessels.^1^ The patient's total cholesterol and LDL cholesterol were significantly high. The xanthomas were consistent with lipid-laden macrophage infiltration, with a clinical presentation of nodular and plaque-like skin lesions on the elbows, knees, popliteal fossae, and palms.

Omega-3 fatty acids, particularly EPA and DHA,^7^ are known for their triglyceride-lowering effects and cardiovascular benefits.^3^ However, DHA can paradoxically raise LDL-C levels,^4^ with studies showing a significant increase of 7.23 mg/dL, while EPA has a milder effect.^5^ In this case, excessive omega-3 intake likely overwhelmed the child’s ability to maintain lipid balance, leading to elevated serum LDL-C and xanthoma formation. According to current guidelines, children aged 4-8 years, the recommended daily intake of omega-3 fatty acids is 900 mg/day, with emphasis on DHA and EPA. The dosage increases to 1200 mg/day in boys aged 9-13 years.^6^ In this case, the child had been consuming 3 or more capsules daily, amounting to 3000-4000 mg, which far exceeded the recommended pediatric dosage.

The primary differential diagnoses for xanthomas in this child included FH, type 3 hyperlipoproteinemia, and drug-induced hypercholesterolemia. FH, a genetic disorder, presents with elevated LDL-C and early-onset xanthomas.^8^ However, the normalization of lipid levels after stopping omega-3 supplementation strongly ruled out FH and type 3 hyperlipoproteinemia.

Previous reports have documented various presentations of pediatric xanthomas. Awal et al described tendinous xanthomas in an 8-year-old girl,^9^ while other studies linked pediatric xanthomas to premature coronary artery disease.^10^

This case highlights the importance of monitoring dietary supplement use in children. While adverse effects of excessive omega-3 fatty acids in children are poorly documented, this case shows the potential for severe lipid disturbances and xanthoma formation. It underscores the need for health education and regulation of over-the-counter supplements.

## Conclusion

A 7-year-old boy with tuberoeruptive and planar xanthomas due to excessive omega-3 supplementation is a rare but instructive example of drug-induced hypercholesterolemia. This case underscores the importance of pediatric-specific guidelines for omega-3 fatty acid consumption and the potential consequences of exceeding recommended dosages.

## Conflicts of interest

None disclosed.
